# Supplementary material for: Physical Exercise Induces Significant Changes in Immunoglobulin G N-Glycan Composition in a Previously Inactive, Overweight Population
Source: Biomolecules. 2023 Apr 27;13(5):762. doi: 10.3390/biom13050762 (PMC10216199; doi:10.3390/biom13050762)
Supplement: Supplementary file 1 [file biomolecules-13-00762-s001.zip › biomolecules-2337978-supplementary.pdf]

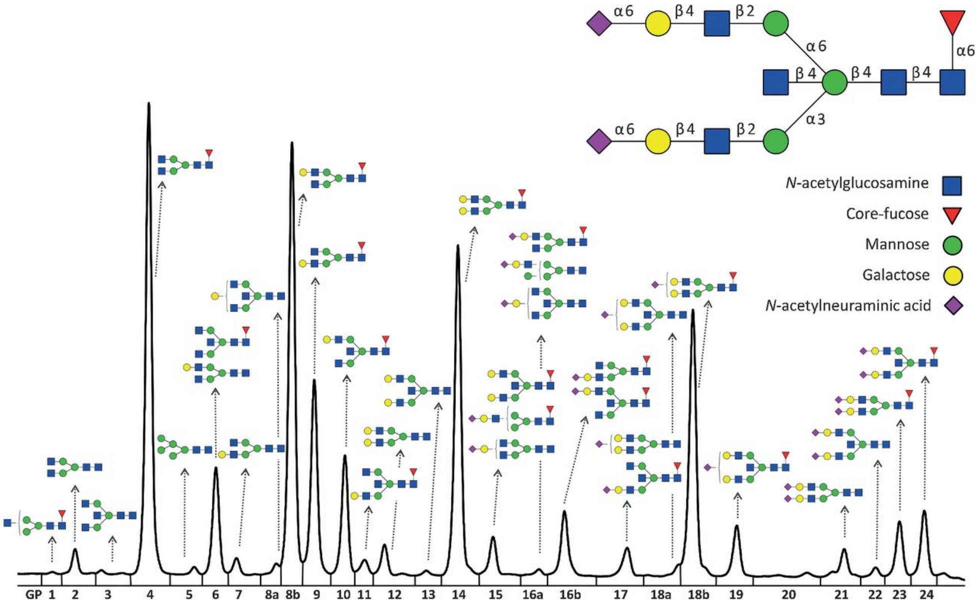

Figure S1. List of IgG N-glycan structures corresponding to individual glycan peaks.

Table S1. Formulae for calculation of IgG-derived glycan traits.

Trait name	Description	Trait formula
G0	Agactosylation	GP1+GP2+GP4+GP6
G1	Monogalactosylation	GP7+GP8+GP9+GP10+GP11+GP16
G2	Digalactosylation	GP12+GP13+GP14+GP15+GP17+GP18+GP19+GP21+GP22+GP23+GP24
S0	Asialylation	GP1+GP2+GP3+GP4+GP5+GP6+GP7+GP8+GP9+GP10+GP11+GP12+GP13+GP14+GP15
S1	Monosialylation	GP16+GP17+GP18+GP19
S2	Disialylation	GP21+GP22+GP23+GP24
B	Bisection	GP6+GP10+GP11+GP13+GP15+GP19+GP22+GP24
CF	Core fucosylation	GP1+GP4+GP6+GP8+GP9+GP10+GP11+GP14+GP15+GP16+GP18+GP19+GP23+GP24

Table S2. Effect (s.d.), standard error (SE) and *p*-values for testing of exercise effect on IgG glycans (adjusted for age and sex). False discovery rate was controlled using Benjamini–Hochberg procedure.

Trait	Effect (s.d.)	SE	*p*	*p* Adjusted
G0	0.044	0.011	5.82×10^{-5}	1.00×10^{-4}
G1	0.248	0.022	4.46×10^{-26}	2.41×10^{-25}
G2	−0.104	0.014	1.70×10^{-12}	4.93×10^{-12}
B	−0.004	0.012	7.41×10^{-1}	7.79×10^{-1}
CF	0.514	0.041	5.42×10^{-31}	3.38×10^{-30}
S0	0.249	0.024	3.54×10^{-22}	1.51×10^{-21}
S1	−0.091	0.015	3.19×10^{-9}	7.61×10^{-9}
S2	−0.526	0.043	1.88×10^{-29}	1.09×10^{-28}
GP1	−0.103	0.041	1.34×10^{-2}	1.78×10^{-2}

GP2	0.033	0.012	7.08×10^{-3}	1.02×10^{-2}
GP3	0.080	0.060	1.86×10^{-1}	2.01×10^{-1}
GP4	0.048	0.011	3.19×10^{-5}	5.63×10^{-5}
GP5	−0.548	0.048	7.69×10^{-26}	3.66×10^{-25}
GP6	0.015	0.010	1.37×10^{-1}	1.54×10^{-1}
GP7	0.052	0.016	1.35×10^{-3}	2.06×10^{-3}
GP8	0.180	0.016	1.19×10^{-24}	5.34×10^{-24}
GP9	0.126	0.014	6.06×10^{-17}	2.05×10^{-16}
GP10	0.065	0.012	4.63×10^{-8}	9.87×10^{-8}
GP11	−0.112	0.024	6.35×10^{-6}	1.17×10^{-5}
GP12	−0.027	0.016	8.61×10^{-2}	1.03×10^{-1}
GP13	0.050	0.048	2.93×10^{-1}	3.12×10^{-1}
GP14	0.032	0.012	7.34×10^{-3}	1.04×10^{-2}
GP15	0.023	0.015	1.33×10^{-1}	1.51×10^{-1}
GP16	0.026	0.017	1.26×10^{-1}	1.46×10^{-1}
GP17	−0.628	0.043	2.18×10^{-38}	1.47×10^{-37}
GP18	−0.025	0.012	4.61×10^{-2}	5.66×10^{-2}
GP19	−0.094	0.027	4.86×10^{-4}	8.03×10^{-4}
GP20	−0.577	0.062	1.50×10^{-18}	5.52×10^{-18}
GP21	−0.925	0.056	2.34×10^{-46}	2.37×10^{-45}
GP22	−0.139	0.052	8.05×10^{-3}	1.12×10^{-2}
GP23	−0.126	0.021	3.63×10^{-9}	8.39×10^{-9}
GP24	−0.095	0.029	1.01×10^{-3}	1.60×10^{-3}
